# Ergosterol Is the Active Compound of Cultured Mycelium *Cordyceps sinensis* on Antiliver Fibrosis

**DOI:** 10.1155/2014/537234

**Published:** 2014-10-15

**Authors:** Yuan Peng, Yanyan Tao, Qinglan Wang, Li Shen, Tao Yang, Zulong Liu, Chenghai Liu

**Affiliations:** ^1^Institute of Liver Diseases, Shuguang Hospital Affiliated to Shanghai University of Traditional Chinese Medicine, 528 Zhangheng Road, Pudong New Area, Shanghai 201203, China; ^2^Shanghai Clinical Key Laboratory of Traditional Chinese Medicine, Shanghai 201203, China; ^3^E-Institute of TCM Internal Medicine, Shanghai Municipal Education Commission, Shanghai 201203, China

## Abstract

Cultured mycelium *Cordyceps sinensis* (CMCS) is a Chinese herbal medicine, which is widely used for a variety of diseases including liver injury in clinic. The current study aims to investigate the protective effects of CMCS against liver fibrosis and to exploit its active antifibrotic substances *in vivo* and *in vitro*. For evaluating the antifibrotic effect of CMCS and ergosterol, male C57BL/6 mice were injected intraperitoneally with carbon tetrachloride (CCl_4_) and treated with CMCS (120 mg/kg/d) or ergosterol (50 mg/kg/d). Four weeks later, serum liver function, hepatic hydroxyproline (Hyp) content, liver inflammation, collagen deposition, and expression of alpha smooth muscle actin (*α*-SMA) in liver tissue were evaluated. Besides, toxicological effects of active compounds of CMCS on hepatocytes and hepatic stellate cells (HSCs) were detected and expressions of permeability of the lysosomal membrane, EdU, F-actin, and *α*-SMA of activated HSCs were analyzed to screen the antifibrotic substance in CMCS *in vitro*. Our results showed that CMCS could significantly alleviate levels of serum liver functions, attenuate hepatic inflammation, decrease collagen deposition, and relieve levels of *α*-SMA in liver, respectively. Ergosterol, the active compound in CMCS that was detected by HPLC, played a dose-dependent inhibition role on activated HSCs via upregulating expressions of permeability of the lysosomal membrane and downregulating levels of EdU, F-actin, and *α*-SMA on activated HSCs *in vitro*. Moreover, ergosterol revealed the antifibrotic effect alike *in vivo*. In conclusion, CMCS is effective in alleviating liver fibrosis induced by CCl_4_ and ergosterol might be the efficacious antifibrotic substance in CMCS *in vivo* and *in vitro*.

## 1. Introduction

Liver fibrosis is a wound-healing response to liver injury that involves a wide range of etiologic agents, including viruses, drugs, metabolic disorders, and immune attacks [1]. Liver fibrosis involves an increased production of extracellular matrix (ECM) components, particularly the deposition of collagens in liver [2]. Activated hepatic stellate cells (HSCs), portal fibroblasts, and myofibroblasts of bone marrow origin have been identified as the major collagen-producing cells in the injured liver [3]. Above all, HSCs, especially the activated HSCs, were identified as the main collagen-producing cells in the liver [4]. In carbon tetrachloride (CCl_4_) model of liver fibrosis, quiescent HSCs are activated to become fibrogenic myofibroblasts [5], which express *α*-smooth muscle actin (*α*-SMA). Since liver fibrosis is closely linked with severe pathological changes including the development of hepatic carcinoma, it is essential to elucidate the mechanisms of restraining the activation and elimination of HSCs, which is considered as a target for the treatment of liver fibrosis [6].

*Cordyceps sinensis* (Berk.) Sacc., as a well-known tonic herb in TCM, is a highly valued fungus in China in regulating the immune function. In recent years, cultured mycelium* Cordyceps sinensis* (CMCS) was successfully developed and widely used as the alternative of natural* Cordyceps sinensis* (Berk.) Sacc. [7]. Accumulated evidences from both animal and human studies indicated that* Cordyceps sinensis* was capable of antifibrosis [8] and anti-inflammation [9]. Whether the active ingredients of CMCS could inhibit HSCs activation and liver fibrosis remained unknown.

In this study, we aimed to elucidate the effects of CMCS on CCl_4_-induced liver fibrosis* in vivo*. Then, we evaluated the effects of the active compounds of CMCS on HSCs activation and hepatic fibrosis* in vitro* and* in vivo*. To clarify our aim, several questions should be formulated. Specifically, they were as follows. (1) Could CMCS inhibit hepatic fibrosis? (2) What were the ingredients of CMCS? (3) Which might be the active antifibrotic compound of CMCS that inhibited HSCs activation and hepatic fibrosis? The results demonstrated that both CMCS and ergosterol, an active ingredient of CMCS, reduced liver injury and hepatic fibrosis. These findings might suggest its potent effects on antifibrosis and exceed its role for designing better antifibrotic drugs.

## 2. Material and Methods

### 2.1. Reagents

CCl_4_ of analytical reagent grade was obtained from Sinopharm Group Co., Ltd. All the other chemicals and solvents used were of analytical grade.

### 2.2. Drug Preparation

The powder of cultured mycelium* Cordyceps sinensis* was purchased from Shanghai Sundise Chinese Medicine Technology Development Co., Ltd. The extraction process of CMCS and determination and quantitation of active compounds in CMCS analyzed by HPLC were all as previously described [10]. Adenosine, ergosterol, vernine, and uridine were all purchased from Shanghai Winherb Medical Science Co., Ltd. The purity of these standards determined by HPLC was more than 98% as per the manufacturer's specification.

### 2.3. Mice

Male C57BL/6 mice (10 weeks of age) were obtained from Shanghai SLAC Laboratory Animal Co. Ltd. (Shanghai, China). Mice were housed in a room under controlled temperature (22–25°C), humidity (46–52%), and lighting (12-hour artificial light and dark cycle), with free access to tap water and mouse chow. The standard diet pellets contained not less than 20% protein, 5% fibers, 3.5% fats, and 6.5% ash and vitamins mixture. Our experiment was conducted according to the local ethical guidelines (Shanghai University of TCM, Shanghai, China).

### 2.4. Induction of Hepatic Fibrosis

For liver fibrosis, mice were intraperitoneally injected with 10% CCl_4_/olive oil 2 mL/kg body weight three times a week, for 4 weeks [11]. Following pentobarbital sodium anesthesia, all mice were sacrificed 24 hours after the last injection. Serum and liver samples were harvested. For histology, tissue specimens were fixed in buffered formalin and embedded in paraffin wax. For fluorescence, liver specimens were embedded in OCT compound. Serum and liver samples were kept frozen at −70°C until assayed.

### 2.5. Animal Groups and Experimental Design

To evaluate the effect of CMCS against liver fibrosis, mice were randomly divided into 4 groups (10 mice per group): normal control group (Normal control), CMCS-treated group (CMCS control), CCl_4_-treated group (Model control), and CCl_4_ plus CMCS-treated group (CMCS treatment). Mice in CMCS control and CMCS treatment were treated orally with CMCS at a daily dose of 120 mg/kg of body weight, respectively, which was equivalent to the dosage of a 60 kg adult. Mice in Normal control and Model control were treated with ddH_2_O by gavage. To investigate the effect of ergosterol against liver fibrosis, mice were randomly divided into 3 groups (10 mice per group): normal control group (Normal control), CCl_4_-treated group (Model control), and CCl_4_ plus ergosterol-treated group (Ergosterol treatment). Mice in Ergosterol treatment were administered orally by gavage with ergosterol at a daily dose of 50 mg/kg of body weight.

### 2.6. Measurements of Serum Liver Function and Hydroxyproline Content

The activities of serum alanine aminotransferase (ALT) and aspartate aminotransferase (AST) and the levels of serum albumin (Alb) and total bilirubin (T.Bil) were quantitated by following the instructions provided by the manufacturer (Nanjing JianCheng Bioengineering Institute, Nanjing, China), including use of standardization. Levels of hydroxyproline (Hyp) in tissue were measured by Jamall's method [12].

### 2.7. Histopathological Assessment of Liver Injury

Liver specimens were fixed in 10% formaldehyde solution and dehydrated in a graded alcohol series, embedded in paraffin blocks, and cut into 4 *μ*m thick slices. For histopathological examination, slices were stained by using standard procedure of hematoxylin and eosin (H&E). For investigation of the hepatic collagen deposition, Sirius red-polarization staining was performed and a red color staining was considered where the collagen deposition. Images were analyzed with light microscope (Olympus BX40, Japan).

### 2.8. Cell Culture

The LX-2 cell line, a spontaneously immortalized human hepatic stellate cell, was a gift from Dr. Scott L. Friedman (Mount Sinai School of Medicine, New York, NY). LX-2 cells were cultured in Dulbecco's modified Eagle's medium (DMEM) supplemented with 10% fetal bovine serum (FBS), 100 units/mL of penicillin, and 100 units/mL of streptomycin. The human hepatic cell line HL-7702, an immortal cell line derived from human adult hepatic tissue, was purchased from Institute of Biochemistry and Cell Biology, SIBS, CAS (Shanghai, China). HL-7702 cells were incubated in RPMI 1640 medium containing 10% heat-inactivated FBS, 100 U/mL penicillin, 100 *μ*g/mL streptomycin, and 2 mM glutamine. All the cells were grown in a humidified incubator at 37°C in a 5% CO_2_ atmosphere.

### 2.9. Drug Treatments

Cells were treated with 6 concentrations (2.5, 5, 10, 20, 40, and 80 *μ*M) of each compound, in duplicate, for a period of 24 h. The compounds were initially dissolved as concentrated stock solutions in dimethyl sulfoxide ([DMSO]; Sigma-Aldrich, USA).

### 2.10. Cell Viability and Proliferation Assay

For HL-7702 cells, cell viability and proliferation assays were performed by using the Cell Counting Kit-8 (CCK8, Beyotime Biotechnology, Jiangsu, China), according to the manufacturer's protocol. For LX-2 cells, cell viability and proliferation assays were detected by Thiazolyl Blue Tetrazolium Blue (MTT, Sigma-Aldrich, USA), according to the manufacturer's protocol. Besides, cytotoxicity of ergosterol on LX-2 was tested by Multiparameter Cytotoxicity II Kit (ThermoFisher Scientific, Germany), aiming to investigate the membrane physical state on lysosomal permeability, and cell proliferation was observed by Cell-Light EdU Apollo 643 DNA* In Vitro* Kit (RiboBio Co. LTD, Guangzhou, China), according to the manufacturer's protocol. Images of the permeability of the lysosomal membrane and EdU were taken by Cellomics ArrayScan VTI HCS Reader (ThermoFisher Scientific, USA) and data were analyzed by Cellomics Cell Health Profiling BioApplication Software.

### 2.11. Immunocytochemistry and Immunofluorescence for *α*-SMA

For immunocytochemistry, LX-2 cells cultured on 96 wells were washed with cold PBS twice and fixed with cold methanol : acetone (1 : 1) for 10 min on ice. After extensive washing with PBS three times, cells were permeated with 0.05% saponin for 15 min. The cells were then blocked with 5% bovine serum albumin in PBS buffer for 30 min at room temperature before being incubated with the alpha smooth muscle actin (*α*-SMA) (1 : 100) primary antibodies (A2547, Sigma, USA). Cells were then stained with FITC-conjugated secondary antibodies. After washing, cells were double-stained with Hoechst 33258 (Beyotime Biotechnology, Jiangsu, China) to visualize the nuclei. Images were taken by Cellomics ArrayScan VTI HCS Reader. For immunofluorescence, liver tissue embedded in OCT compound was cut into 9 micrometers thick and fixed in 4% formaldehyde (Dingguo, Shanghai, China). After washing with PBS for three times, sections were permeabilized with 0.1% Triton X-100 and blocked with 5% BSA in 0.1 M PBS for 20 minutes at room temperature to reduce nonspecific binding and then were incubated with primary antibodies against *α*-SMA (M0851, DAKO, Japan) followed by the biotinylated secondary antibody. To visualize the primary antibodies, cells were stained with FITC-conjugated secondary antibodies. After washing, cells were double stained with 4′,6-diamidino-2-phenylindole (DAPI) to visualize the nuclei. Images were obtained using a confocal microscope and photographs were taken with Olympus confocal software. The semiquantification data for *α*-SMA level in the liver tissue were determined with the computer-assisted image analysis system.

### 2.12. F-Actin Cytoskeleton Staining

LX-2 cells were grown in a 96-well chamber and treated with 5 ng/mL transforming growth factor-beta1 (TGF-*β*1) and drugs. After 24 hours, cells were fixed in 4% formaldehyde and permeabilized with 0.2% triton X-100. F-actin was stained with rhodamine phalloidin (1 : 100) (Molecular Probes, Inc., Eugene, Oregon, USA) and the nucleus with DAPI, according to the manufacturer's protocol. Cells were visualized by Cellomics ArrayScan VTI HCS Reader and data were analyzed by Cellomics Cell Health Profiling BioApplication Software.

### 2.13. Statistical Analysis

All data were analyzed by using PASW Statistics 18 software. Differences between the groups were assessed by nonparametric one-way analysis of variance (ANOVA) followed by the least significant difference (LSD) post hoc tests. Values in the text are means ± standard deviation (SD). Differences with *P* < 0.05 were considered to be statistically significant.

## 3. Results 

### 3.1. CMCS Attenuated CCl_4_-Induced Liver Fibrosis in Mice

**Figure 1:**
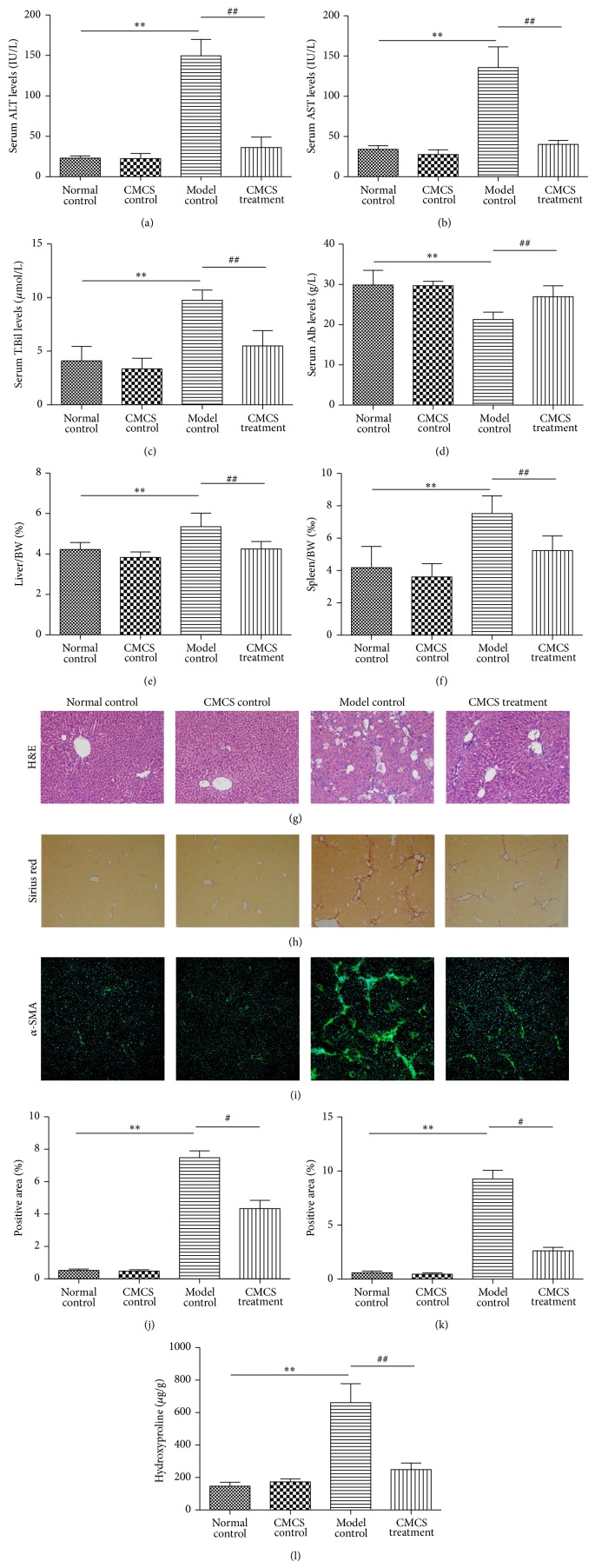
Antifibrotic effects of CMCS on CCl_4_-induced liver fibrosis in mice. Mice of ten-week old were treated as described in the legend. Serum ALT (a), AST (b), Alb (c), and T.Bil (d) were assessed by liver function tests kits, respectively. Levels of serum liver functions were decreased, and liver/BW (e) and spleen/BW (f) were alleviated by CMCS treatment in CCl_4_-induced liver fibrosis mice. Histologic evaluation of liver tissues were stained with H&E ((g), ×200). Collagen deposition was revealed with Sirius Red staining ((h), ×100) and was shown as the proportion of Sirius red-positive area (j). Expressions of *α*-SMA in liver tissues were analyzed by immunofluorescent staining. Representative bright-field and fluorescent micrographs were shown ((i), ×100). Semiquantification of *α*-SMA expression was evaluated and shown as percentage of *α*-SMA-positive areas (k). Hydroxyproline content was quantified from 100 mg liver samples and was measured by Jamall's method (l). ^*^ *P* < 0.05, ^**^ *P* < 0.001, versus Normal control; ^#^ *P* < 0.05, ^##^ *P* < 0.001, versus Model control.

In order to evaluate the effect of CMCS on CCl_4_-induced liver fibrosis, levels of serum liver functions were assayed by kits. Levels of serum ALT, AST, and T.Bil and higher levels of serum Alb were lower in Normal control compared with those in Model control (Figures 1(a), 1(b), 1(c), and 1(d)). The levels of ALT, AST, and T.Bil were significantly reduced in CMCS-treated mice. Besides, CMCS alleviated the ratios of liver/body weight (BW) (%) and spleen/BW (*‰*) in CCl_4_-induced hepatic fibrosis mice, respectively (Figures 1(e) and 1(f)). For investigating the inflammation and collagen accumulation in the liver tissue, H&E and Sirius Red staining were observed, respectively (Figures 1(g) and 1(h)). In Normal control and CMCS control groups, the hepatocytes were radially arranged around a central vein with no collagen fibers present, respectively. In Model control, hepatic plates were diffusely disrupted with pronounced ballooned/necrotic hepatocytes containing acidophilic hyaline inclusions, which were degenerated with widely mononuclear cell infiltration present in the portal area and fibrous septa (Figure 1(g)). Hepatic parenchyma was expanded by collagen fibers, and lobules were separated by fibrous septa that surrounded the hepatocytes (Figure 1(h)). Hepatic inflammation and fibrosis were significantly alleviated by CMCS treatment, as evidenced by reduced inflammatory cell infiltration and thinner fibrous septa (Figures 1(h) and 1(j)). Similarly, hepatic hydroxyproline content was dropped by CMCS treatment in model mice (Figure 1(l)).

### 3.2. CMCS Reduced Expression of *α*-SMA* In Vivo*

Since CMCS could attenuate CCl_4_-induced liver fibrosis, we next examined the effect of CMCS on the expression of hepatic *α*-SMA, the gold marker of liver fibrosis and HSCs activation. In Normal control and CMCS control group, positive staining for *α*-SMA was mainly observed in vascular walls of central veins and portal regions, while the stronger *α*-SMA staining was seen in Model control, especially in the vicinity of the bile ductules and the fibrous septa (Figures 1(i) and 1(l)). The expression of *α*-SMA was significantly decreased in CMCS treatment group, compared with that in Model control (Figures 1(i) and 1(l)). These results revealed that CMCS could significantly inhibit the activation of HSCs.

### 3.3. Ergosterol Inhibited Activities of HSCs* In Vitro*

**Figure 2:**
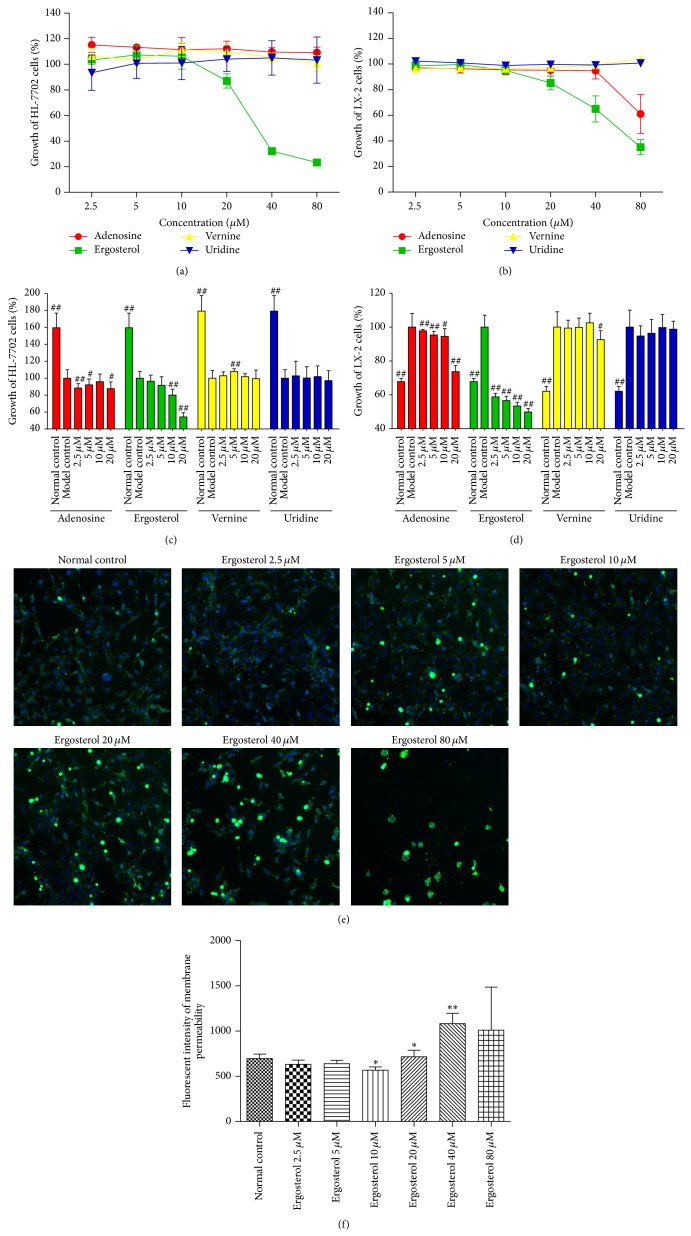
Cytotoxicity and proliferation of adenosine, ergosterol, vernine, and uridine in HL-7702 and LX-2 cell lines. ((a) and (b)) Cells were cultured in a 96-well plate at a density of 4,000 cells/well and incubated with 2.5–80 *μ*M of adenosine, ergosterol, vernine, and uridine for 24 h, respectively. For HL-7702 cell, cytotoxicity was detected by CCK8. For LX-2 cell, cytotoxicity was analyzed by MTT. Representative dose-response curves in HL-7702 cell (a) and LX-2 cell (b) culture with adenosine, ergosterol, vernine, and uridine for 24 h were represented by line chart, respectively. ((c) and (d)) Cells were cultured in a 96-well plate at a density of 4,000 cells/well. HL-7702 cells were exposed with 0.5 mM H_2_O_2_ for 30 min and LX-2 cells were incubated with 0.5 ng/mL TGF-*β*1 for 24 h. The following cells were cultured with 2.5–20 *μ*M adenosine, ergosterol, vernine, and uridine for 24 h, respectively. For HL-7702 cell, proliferation was detected by CCK8. For LX-2 cell, proliferation was analyzed by MTT. Representative dose-response curves in HL-7702 cell (c) and LX-2 cell (d) were represented in comparison with the Model control (100%) and were shown as growth of the cells, respectively. (e) LX-2 cells were cultured in a 96-well plate at a density of 4,000 cells/well. Cells were incubated with 2.5–80 *μ*M of ergosterol for 24 h. Images of permeability of the lysosomal membrane were investigated by Cellomics ArrayScan VTI HCS Reader. The nuclear and lesioned lysosomal membranes were stained with blue and green, respectively. (f) The image analysis of immunofluorescence used by Cellomics Cell Health Profiling BioApplication Software. ^*^ *P* < 0.05, ^**^ *P* < 0.01, versus Normal control. ^#^ *P* < 0.05, ^##^ *P* < 0.01, versus Model control.

**Figure 3:**
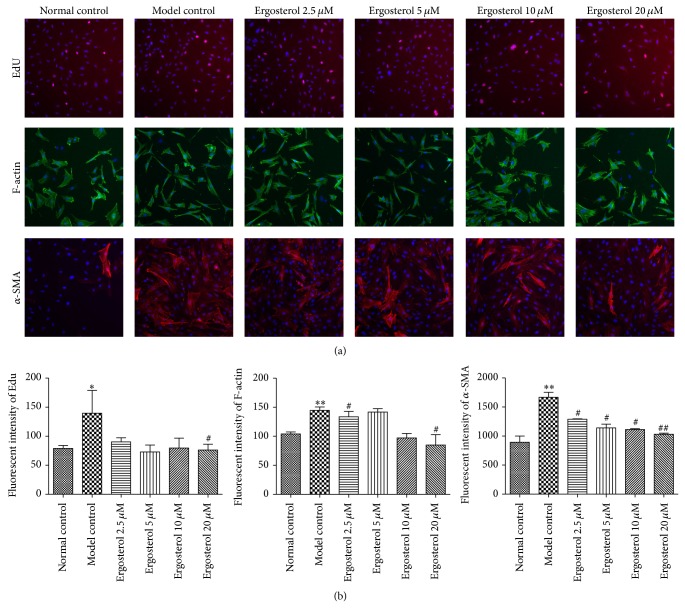
Effects of ergosterol on expressions of EdU, F-actin, and *α*-SMA induced by TGF-*β*1 in LX-2 cell. (a) LX-2 cells were cultured in a 96-well plate at a density of 4,000 cells/well. Cells were stimulated by 5 ng/mL TGF-*β*1 for 24 h and then incubated with 2.5–20 *μ*M of ergosterol for 24 h. Images of EdU, F-actin, and *α*-SMA were investigated by Cellomics ArrayScan VTI HCS Reader, respectively. The nuclear and proliferating cells were stained with blue and pink, respectively. (b) The image analysis of immunofluorescence used by Cellomics Cell Health Profiling Bio Application Software. ^*^ *P* < 0.05, ^**^ *P* < 0.01, versus Normal control; ^#^ *P* < 0.05, ^##^ *P* < 0.05, versus Model control.

We previously detected the active compounds of CMCS by HPLC, finding that CMCS mainly contained four ingredients: adenosine, ergosterol, vernine, and uridine. In an effort to define the potential active substances of CMCS that might play the critical roles in resisting liver fibrosis, we observed the effects of these four monomers on human hepatocytes and HSC cell lines* in vitro*, respectively. Firstly, HL-7702 and LX-2 cells were preincubated with 2.5–80 *μ*M of adenosine, ergosterol, vernine, and uridine for 24 h, respectively. After 24 h, the survival ratio of HL-7702 and LX-2 cells in incubations with 2.5–20 *μ*M of adenosine, ergosterol, vernine, and uridine for 24 h exceeded 80%, respectively (Figures 2(a) and 2(b)), which showed no obvious toxic to the cells (Table 1). Secondly, HL-7702 cells were exposed to 0.5 mM H_2_O_2_ for 30 min to induce hepatocellular injury* in vitro*. Subsequently, cells were incubated with 2.5–20 *μ*M adenosine, ergosterol, vernine, and uridine for 24 h. 24 hours later, the four active ingredients of CMCS were not able to protect against hepatocellular injury induced by H_2_O_2_ *in vitro* (Figure 2(c)). Finally, LX-2 cells were incubated with 5 ng/mL TGF-*β*1 for 24 h to induce HSC activation. Then, cells were cultured with 2.5–20 *μ*M adenosine, ergosterol, vernine, and uridine for 24 h, respectively. 24 hours later, HSCs activation could not be restrained by the other monomers of CMCS except ergosterol (Figure 2(d)). Surprisingly, ergosterol had a dose-dependent inhibition on HSCs activation* in vitro*, which revealed that ergosterol might be able to act a vital role in resisting liver fibrosis* in vitro*. To confirm the antifibrotic effect of ergosterol* in vitro*, we proceeded to explore the effect of ergosterol on permeability of the lysosomal membrane in LX-2 cells. As expected, the permeability of the lysosomal membrane in LX-2 cells was altered by treatment with ergosterol for 24 h (Figures 2(e) and 2(f)). Incubating with 10 *μ*M ergosterol decreased the level of permeability of the lysosomal membrane but increased in the concentration of 20 *μ*M (Figures 2(e) and 2(f)). Besides, proliferation of activated LX-2 cells stimulated by 5 ng/mL TGF-*β*1 for 24 h was inhibited after treatment with 10 *μ*M ergosterol for another 24 h unsurprisingly (Figure 3). In addition, cytoskeleton protein expression of LX-2 cells was significantly downregulated and levels of *α*-SMA showed a decreasing trend by cultured with ergosterol* in vitro*, respectively, which further proved that ergosterol, as a highly efficacious substance in CMCS, could inhibit HSCs activation* in vitro* (Figure 3).

### 3.4. Ergosterol Protected Liver against CCl_4_-Induced Hepatic Fibrosis* In Vivo*

**Figure 4:**
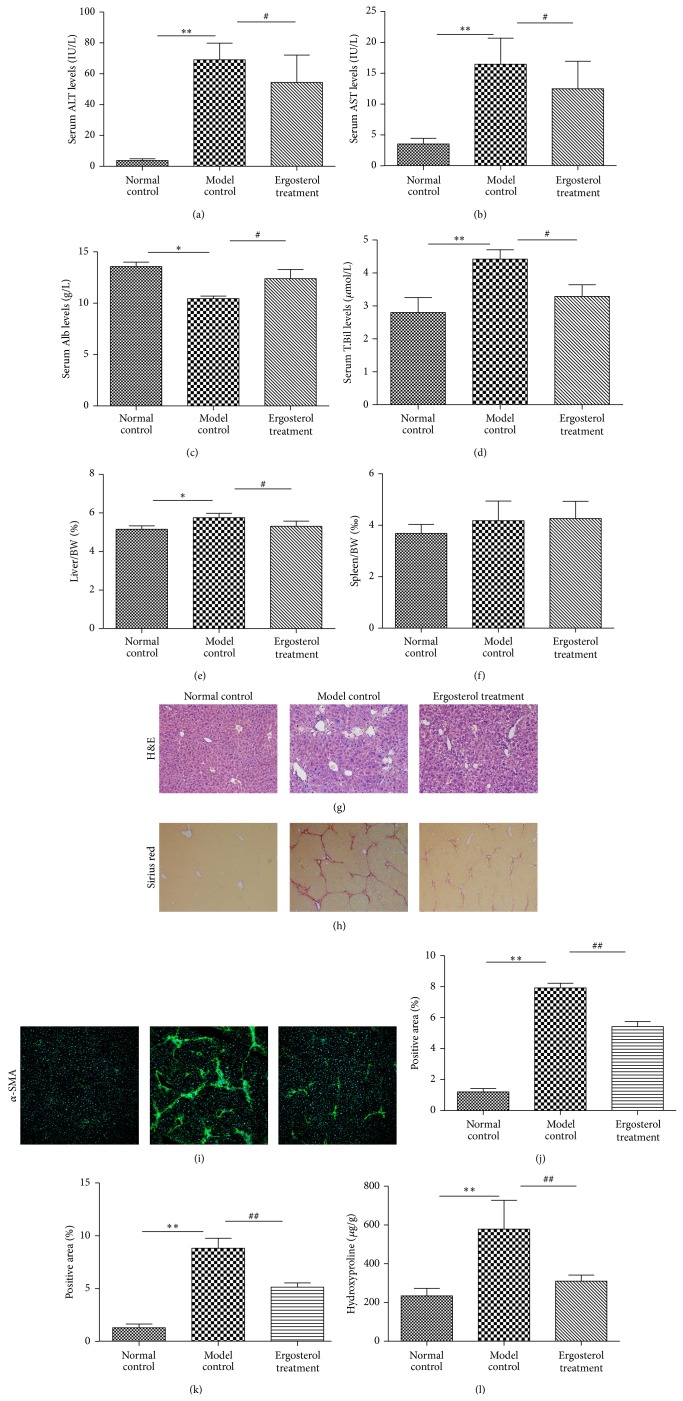
Antifibrotic effects of ergosterol on CCl_4_-induced liver fibrosis in mice. Mice of ten-week old were treated as described in the legend. Serum ALT (a), AST (b), Alb (c), and T.Bil (d) were assessed by liver function tests kits, respectively. Levels of serum liver functions were decreased, and ratios of liver/BW (e) and spleen/BW (f) were alleviated by Ergosterol treatment in CCl_4_-induced liver fibrosis mice. Histologic results of liver tissues were stained with H&E ((g), ×200). Quantification of the same three groups in (g) with respect to Sirius Red staining ((h), ×100). Collagen deposition was shown as percentage of Sirius red-positive area (j). Liver tissues were analyzed by staining for *α*-SMA immunofluorescence. Representative bright-field and fluorescent micrographs were shown ((i), ×100). Semiquantification data for *α*-SMA expressions in the liver tissue were evaluated and were shown as percentage of *α*-SMA-positive areas (k). Hydroxyproline content was quantified from 100 mg liver samples and measured by Jamall's method (l). ^**^ *P* < 0.01, versus Normal control; ^##^ *P* < 0.01, versus Model control.

Treatment of intragastric administration with 50 mg/kg ergosterol was given to investigate the prevention of liver fibrosis induced by 10% CCl_4_ *in vivo*. Model control group had much higher serum levels of ALT, AST, T.Bil, liver/BW, and spleen/BW and lower Alb than those of the Normal control group (Figures 4(a), 4(b), 4(c), 4(d), 4(e), and 4(f)). Furthermore, more severe liver inflammation, collagen deposition, and expression of *α*-SMA were seen in the Model control group, respectively (Figures 4(g), 4(h), 4(i), and 4(j)), compared to those in the Normal control group. Ergosterol treatment ameliorated serum liver function, decreased liver/BW or spleen/BW, attenuated hepatic inflammation, decreased collagen deposition, and improved expression of *α*-SMA in Model group, which obviously indicated that ergosterol could protect against hepatic fibrosis* in vivo*.

## 4. Discussion

Liver fibrosis represents chronic wound repair following liver injury [13]. Upon liver injury, quiescent HSCs, the most relevant cell type for hepatic fibrogenesis, become active, convert into myofibroblast-like cells, and overproduce extracellular matrix (ECM) [14], which actually worsen the liver damage. The terminal outcome of liver fibrosis is the formation of nodules encapsulated by fibrillar scar matrix [15]. Therefore, it is imperative that inhibition of HSCs activation is urgently needed.

Natural* Cordyceps sinensis* (Berk.) Sacc has been widely used in China for potentially vulnerable people with chronic diseases in all ages, and its use is strongly supported by clinical application. Nowadays, CMCS is widely used as the alternative of natural* Cordyceps sinensis* (Berk.) Sacc. [7], which has better curative effect and lightens the economic burden of patients in low income group. CMCS contains an array of active substances, which reveal the advantage of potential synergy between the various components. More and more research information has convinced its significant pharmacological properties. Some CMCS related nutraceuticals available in the market are considered to relieve the “stress for humans of living in technologically developed societies” by stimulating basic and secondary responses of the immune system [16].

To explore the antifibrotic effect on CMCS, we have investigated the role of CMCS and its active compounds in hepatic fibrosis* in vivo* and* in vitro*. In our study, we firstly confirmed earlier findings that the extractum of CMCS could alleviate levels of serum liver function in CCl_4_-induced liver fibrosis mice* in vivo*. Then, we further analyzed the pathology of liver tissue to evaluate the efficacy of CMCS on treatment of liver fibrosis. It was obvious that CMCS could relieve the infiltration of inflammatory cells and collagen deposition. Besides, expression of *α*-SMA, the golden marker of activated HSC, was decreased by CMCS treatment. To observe the toxicity of CMCS, normal mice were administrated with the same dosage of CMCS in the meantime. The results of serum liver function and histopathological assessment indicated that treatment with CMCS for four weeks remained no hepatotoxicity for normal mice. These results suggested that CMCS had potential antifibrotic effect on CCl_4_-induced liver fibrosis.

We previously made a quantitative analysis on the freeze drying powder of CMCS extractum by HPLC to detect the main active compounds. Subsequently, our TCM fingerprint revealed that CMCS mainly contained four active ingredients: adenosine, ergosterol, vernine, and uridine. Herein, we observed the toxicological and pharmacological studies on these four monomers on HSCs (LX-2 cell lines)* in vitro* in order to discover the main potential active substances in CMCS that played critical roles in resisting liver fibrosis. Moreover, the active antifibrotic substance should be with no hepatotoxicity. Hence, toxicological study of the four monomers was performed on hepatocytes (HL-7702 cell lines)* in vitro* simultaneously. The results of cytotoxicity and proliferation in HL-7702 and LX-2 implied that ergosterol had no hepatotoxicity on hepatocytes. In addition, it took effects in manner of dose-dependent inhibition on activated HSCs* in vitro*.

Lysosomes are involved in the cellular degradation of material either initially present in the cell or brought into the cell by phagocytosis or endocytosis [17]. Lysosomal destabilization is critical for the organelle and living cells [18]. In our study, we found ergosterol could decrease the level of permeability of the lysosomal membrane for HSCs at a low concentration, which revealed that ergosterol might be liable for damage on cell membrane of HSCs* in vitro*. Evidently, permeation of EdU and expression of *α*-SMA of HSCs stimulated by TGF-*β* were repressed by ergosterol at a concentration of 20 *μ*M* in vitro*, respectively. In nonmuscular cells, F-actin forms a system of fibers that provide mechanical support for several structures and, in association with myosin, assembles contractile units responsible for cellular movements. In our study, the expression of F-actin was increased in HSCs stimulated by TGF-*β* while it was decreased after ergosterol intervention, which indicated that ergosterol might disrupt the cytoskeleton of HSCs.

In the following studies* in vivo*, ergosterol, like CMCS, could ameliorate serum liver function, attenuate hepatic inflammation, decrease collagen deposition, and improve the expression of *α*-SMA in mouse model of liver fibrosis, respectively, which obviously confirmed that ergosterol could protect liver against hepatic fibrosis* in vivo*. This finding was conformed to that of experiments* in vivo*.

Our results demonstrated that CMCS and ergosterol attenuate hepatic fibrosis induced by CCl_4_ in mice. We confirmed the pharmacological effects of CMCS on inhibiting the active HSCs. We further defined that the antifibrotic effect of ergosterol was associated with the restriction of proliferation, wreck of cytoskeleton, and activation of HSCs. Our study is the first to report that ergosterol is the active compound of CMCS on antiliver fibrosis. However, as a therapeutic agent, the accurate effect of CMCS and ergosterol's effect on restraining liver fibrosis still need to be investigated. Further studies and other animal models of liver fibrosis are necessary to elucidate the precise mechanisms of their antifibrosis effects.

## 5. Conclusions

CMCS is effective in alleviating liver fibrosis induced by CCl_4_ *in vivo* and ergosterol might be the efficacious antifibrotic substance of CMCS* in vivo* and* in vitro*, which might provide alternative treatment options for hepatic fibrosis.

## Figures and Tables

**Table 1:** IC_50_ values (*μ*M) of active compounds in CMCS^a^.

Cell line	Active compounds			
	Adenosine	Ergosterol	Vernine	Uridine
HL-7702	nc	37.10 (±2.07)	nc	nc
LX-2	nc	55.51 (±7.13)	nc	nc

^a^Values are means of at least three experiments; standard deviation is given in parentheses (nc = no toxic).

## Abbreviations

CMCS: Cultured mycelium* Cordyceps sinensis*
CCl_4_: Carbon tetrachloride
ALT: Alanine aminotransferase
AST: Aspartate aminotransferase
Alb: Albumin
T.Bil: Total bilirubin
Hyp: Hydroxyproline
H&E: Hematoxylin and eosin
*α*-SMA: Alpha smooth muscle actin
HPLC: High performance liquid chromatography
CCK8: Cell Counting Kit-8
MTT: Thiazolyl Blue Tetrazolium Blue
HSCs: Hepatic stellate cells
DMEM: Dulbecco’s modified Eagle’s medium
FBS: Fetal bovine serum
DMSO: Dimethyl sulfoxide
BSA: Bovine plasma albumin
PBS: Phosphate-buffer saline
DAB: 3,3′-Diamino Benzedrine
DAPI: 4′,6-Diamidino-2-phenylindole
TGF-*β*: Transforming growth factor-beta
BW: Body weight.

## References

[1] Friedman S. L. (2008). Mechanisms of hepatic fibrogenesis. *Gastroenterology*.

[2] Ballin M., Gomez D. E., Sinha C. C., Thorgeirsson U. P. (1988). Ras oncogene mediated induction of a 92kDa metalloproteinase; strong correlation with the malignant phenotype. *Biochemical and Biophysical Research Communications*.

[3] Bataller R., Brenner D. A. (2005). Liver fibrosis. *The Journal of Clinical Investigation*.

[4] Friedman S. L., Roll F. J., Boyles J., Bissell D. M. (1985). Hepatic lipocytes: the principal collagen-producing cells of normal rat liver. *Proceedings of the National Academy of Sciences of the United States of America*.

[5] Kisseleva T., Cong M., Paik Y., Scholten D., Jiang C., Benner C., Iwaisako K., Moore-Morris T., Scott B., Tsukamoto H., Evans S. M., Dillmann W., Glass C. K., Brenner D. A. (2012). Myofibroblasts revert to an inactive phenotype during regression of liver fibrosis. *Proceedings of the National Academy of Sciences of the United States of America*.

[6] Bataller R., Brenner D. A. (2001). Hepatic stellate cells as a target for the treatment of liver fibrosis. *Seminars in Liver Disease*.

[7] Shen Xiaoyun L. Z., Jun T. (1998). Chemical component comparison between *Cordyceps sinensis* and Mycelia of *Cordyceps sinensis*. *Journal of Shanxi University*.

[8] Nan J. X., Park E. J., Yang B. K., Song C. H., Ko G., Sohn D. H. (2001). Antifibrotic effect of extracellular biopolymer from submerged mycelial cultures of *Cordyceps militaris* on liver fibrosis induced by bile duct ligation and scission in rats. *Archives of Pharmacal Research*.

[9] Won S.-Y., Park E.-H. (2005). Anti-inflammatory and related pharmacological activities of cultured mycelia and fruiting bodies of *Cordyceps militaris*. *Journal of Ethnopharmacology*.

[10] Peng Y., Chen Q., Yang T., Tao Y., Lu X., Liu C. (2014). Cultured mycelium *Cordyceps sinensis* protects liver sinusoidal endothelial cells in acute liver injured mice. *Molecular Biology Reports*.

[11] Radaeva S., Sun R., Jaruga B., Nguyen V. T., Tian Z., Gao B. (2006). Natural killer cells ameliorate liver fibrosis by killing activated stellate cells in NKG2D-dependent and tumor necrosis factor-related apoptosis-inducing ligand-dependent manners. *Gastroenterology*.

[12] Jamall I. S., Finelli V. N., Que Hee S. S. (1981). A simple method to determine nanogram levels of 4-hydroxyproline in biological tissues. *Analytical Biochemistry*.

[13] Friedman S. L. (1999). Cytokines and fibrogenesis. *Seminars in Liver Disease*.

[14] Friedman S. L. (2000). Molecular regulation of hepatic fibrosis, an integrated cellular response to tissue injury. *The Journal of Biological Chemistry*.

[15] Tsukamoto H. (1999). Cytokine regulation of hepatic stellate cells in liver fibrosis. *Alcoholism: Clinical and Experimental Research*.

[16] Lakhanpal T. N., Rana M. (2005). Medicinal and nutraceutical genetic resources of mushrooms. *Plant Genetic Resources: Characterisation and Utilisation*.

[17] Docherty K., Maguire G. A., Hales C. N. (1983). Permeability properties of lysosomal membranes. *Bioscience Reports*.

[18] Hao S.-J., Hou J.-F., Jiang N., Zhang G.-J. (2008). Loss of membrane cholesterol affects lysosomal osmotic stability. *General Physiology and Biophysics*.

